# Estimate of overdiagnosis of breast cancer due to mammography after adjustment for lead time. A service screening study in Italy

**DOI:** 10.1186/bcr1625

**Published:** 2006-12-05

**Authors:** Eugenio Paci, Guido Miccinesi, Donella Puliti, Paola Baldazzi, Vincenzo De Lisi, Fabio Falcini, Claudia Cirilli, Stefano Ferretti, Lucia Mangone, Alba Carola Finarelli, Stefano Rosso, Nereo Segnan, Fabrizio Stracci, Adele Traina, Rosario Tumino, Manuel Zorzi

**Affiliations:** 1 Clinical and Descriptive Epidemiology Unit, CSPO, Research Institute of the Tuscany Region, via di San Salvi 12, Firenze, 50135, Italy; 2 ASL Bologna Area Nord, via Libertà 45, 40016, San Giorgio di Piano, Bologna, Italy; 3 U.O. Oncologia, Azienda Ospedaliera-Università, Parma Cancer Registry, via Abbeveratoia 4, Parma, 43100, Italy; 4 Dipartimento Interaziendale di Oncologia, Ospedale G.B. Morgagni–L. Pierantoni, Pad. Valsava, Romagna Cancer Registry, via C. Forlanini 34, Forlì, 47100, Italy; 5 Modena Cancer Registry, via del Pozzo 71, Modena 41100, Italy; 6 Ferrara Cancer Registry, Dipartimento di Medicina Sperimentale e Diagnostica, Sezione di Anatomia, Istologia e Citologia Patologica, Università di Ferrara, v. Fossato di Mortara 64B, Ferrara, 44100, Italy; 7 Epidemiology Unit, Reggio-Emilia Cancer Registry, via Amendola 2, Reggio-Emilia, 42100, Italy; 8 Screening Programme, Emilia-Romagna Region Health Department, viale Aldo Moro 21, 40127, Bologna, Italy; 9 CPO – Piedmont Cancer Registry, via San Francesco da Paola 31, Torino,10123, Italy; 10 CPO Piemonte, Epidemiology Unit, via San Francesco da Paola 31, Torino, 10123, Italy; 11 Umbria Cancer Registry, Dipartimento di specialità Medico-Chirurgiche e Sanità Pubblica, Università degli Studi di Perugia, via del Giochetto, Perugia, 06100, Italy; 12 Department of Oncology, ARNAS Ascoli, via Parlavechio 1, 90100, Palermo, Italy; 13 Department of Oncology 'Sebastiano Ferrara', Cancer Registry andUnit of Histopathology Azienda Ospedaliera 'Civile M.P. Arezzo', via Dante 109, Ragusa, 97100, Italy; 14 Dipartimento di Scienze oncologiche e chirurgiche, Università degli Studi di Padova, Istituto Oncologico Veneto (IOV), via Gattamelata 64, Padova, 35128, Italy

## Abstract

**Introduction:**

Excess of incidence rates is the expected consequence of service screening. The aim of this paper is to estimate the quota attributable to overdiagnosis in the breast cancer screening programmes in Northern and Central Italy.

**Methods:**

All patients with breast cancer diagnosed between 50 and 74 years who were resident in screening areas in the six years before and five years after the start of the screening programme were included. We calculated a corrected-for-lead-time number of observed cases for each calendar year. The number of observed incident cases was reduced by the number of screen-detected cases in that year and incremented by the estimated number of screen-detected cases that would have arisen clinically in that year.

**Results:**

In total we included 13,519 and 13,999 breast cancer cases diagnosed in the pre-screening and screening years, respectively. In total, the excess ratio of observed to predicted *in situ *and invasive cases was 36.2%. After correction for lead time the excess ratio was 4.6% (95% confidence interval 2 to 7%) and for invasive cases only it was 3.2% (95% confidence interval 1 to 6%).

**Conclusion:**

The remaining excess of cancers after individual correction for lead time was lower than 5%.

## Introduction

Breast cancer service screening programmes have been implemented on a regional basis in several Italian areas. Most screening programmes are participants in the national survey promoted by the Italian Group for Mammography Screening [1] and have collected performance data in accordance with the European guidelines for breast cancer screening [2,3].

Early diagnosis of breast cancer and excess incidence are the expected consequences of breast cancer screening. The possible detection at screening of breast cancers that would not have been diagnosed in the absence of screening over a subject's lifetime has been defined as overdiagnosis [4,5].

In this paper we present observational data from a large Italian study – the Impact Study – in which we apply a statistical model for monitoring service screening to forecast the possible occurrence of overdiagnosis. This study is based on the evaluation of individual cases and not on aggregated data, which have been used in most studies evaluating service screening so far.

## Materials and methods

### The Impact Study

The Impact Study included breast cancers diagnosed between 1986 and 2001 in women aged 40 to 79 years who were resident in 17 areas mainly located in Central and Northern Italy. Breast cancers were included in accordance with the International Agency for Research on Cancer rules for cancer registration [6]. *In situ *carcinomas were included, and death certificate only (DCO) cases were excluded. All registry-based breast cancer cases were linked to the screening file and divided up by detection method. We classified cases as either screen detected or not screen detected, and as invited or uninvited to screening.

In this paper we have included areas with at least five years of screening data: Torino, Parma, Ferrara, Modena, Romagna and Firenze (Table 1). *In situ *carcinomas were missing in a few pre-screening years in two registries. In these cases, the incidence of *in situ *carcinoma was estimated from age-specific data of the two subsequent pre-screening years. Incidence rates were calculated from data on populations by area, year and age class produced by the National Statistics Institute.

The pooled annual trend of incidence in the pre-screening period was modelled through a two-step Poisson analysis process. In the first step, a Poisson regression model (model 1) including age (annual, range from 40 to 79 years) and calendar time (continuous, year) was fitted to the available pre-screening incidence data for each area. Interaction terms between age and calendar period were not found statistically significant in any area (*P *= 0.05). In the second step, a pooled Poisson model (model 2) including age (continuous), calendar time (continuous, year) and area as independent variables, based on observed and estimated by model 1, predicted rates for each area for the period 1986 to 2001. Throughout this paper we have presented time on a screening time scale, where 1 is the first year of the screening programme. We consider two periods: six years of pre-screening (years -5 to 0) and five years of screening (years 1 to 5).

### Screening process and correction for lead time

Screening works by detecting breast cancer in the early phase of its natural history; the period during which a tumour is in the pre-clinical detectable phase is known as its sojourn time. In several studies an estimate of sojourn time has been fitted with an exponential distribution [7]. Following this, the estimated mean sojourn time (MST) is 1/λ, where λ is the average of the exponential distribution (that is, the rate of progression from the preclinical to the clinical phase, as estimated from screening data).

**Figure 1:**
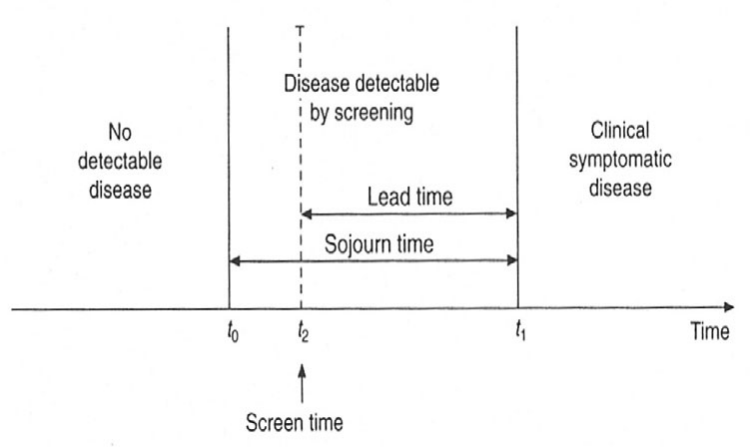
Natural history of breast cancer and screening.

Given these assumptions, the MST is also the estimate of the average lead time of screen-detected cases. It is therefore possible to estimate the probability that a tumour currently detected at screening in the pre-clinical phase would have of progressing to the clinical phase in each year after detection in the absence of screening. If we suppose that a breast cancer is diagnosed at time *t*_2 _in the preclinical detectable phase (see Figure 1), then it is possible to estimate the probability that the case would have of surfacing as symptomatic each year afterwards. Some screen-detected cases will be expected to arise in the short term (that is, they have short lead time) and others in the long term (a long lead time).

Thus it is possible to calculate the probability that each screen-detected case would have been identified clinically each year after detection until a defined time, such as the end of the study period. The sum of these probabilities over all the screen-detected cases, year by year, gives an estimate of the number of screen-detected cases that would have arisen clinically each year. The corrected-for-lead-time number of observed cases for each calendar year corresponds to the number of observed incident cases reduced by the number of screen-detected cases in that year, and incremented by the estimated number of screen-detected cases (in whatever year) that would have arisen clinically in that year [8]. Following the terminology suggested by Etzioni [9], the number of incremental cases (that is, the number of screen-detected cases) should be compensated for by the number of decremental cases (that is, the number of screen-detected cases that would have arisen clinically). In a certain number of years these figures should equalise (assuming no overdiagnosis). The corrected-for-lead-time cases should be compared with the predicted number in the absence of screening, and the percentage excess after correction for lead time is an indicator of overdiagnosis, given the lead time estimate.

In screening literature, there is quite a good agreement between estimates of the mean sojourn time of breast cancer: between three and four years, and longer at higher ages. In this paper we modelled with MST durations of 3.7 and 4.2 years for women aged 50 to 59 years and 60 to 74 years at screen detection, respectively [10].

**Figure 2:**
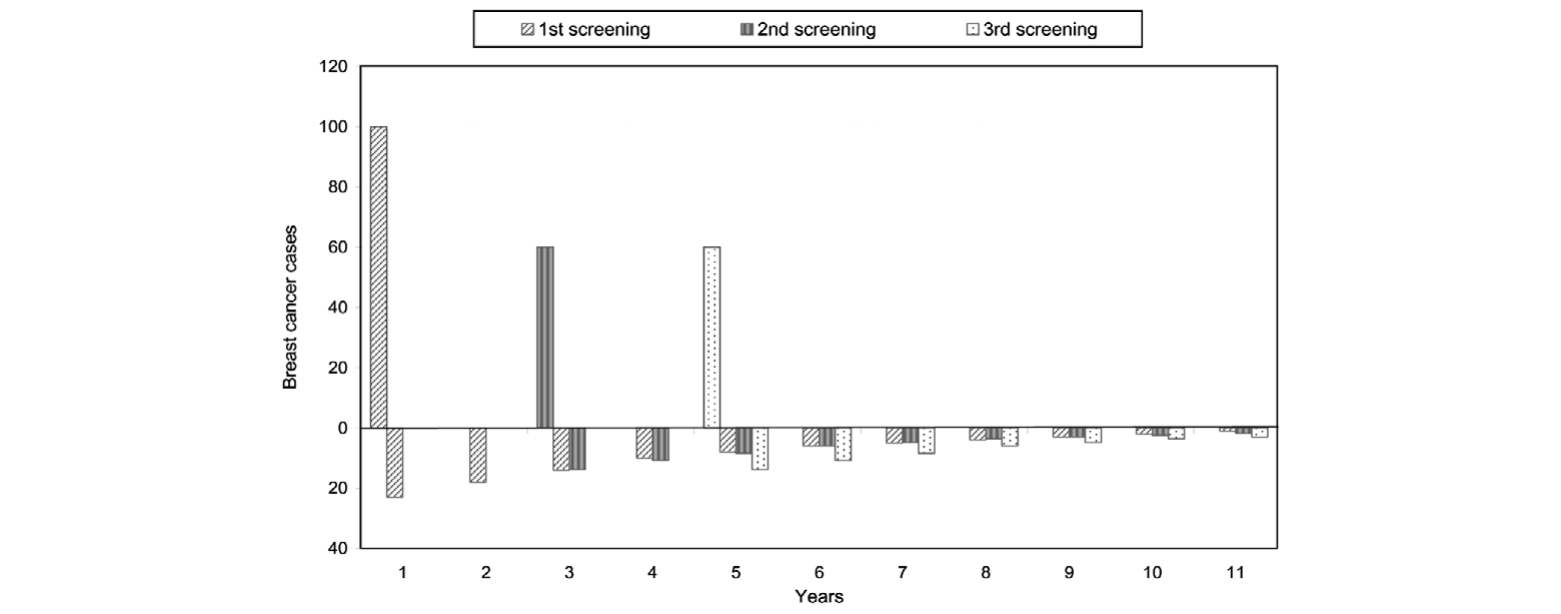
Incremental and decremental cases in a cohort of women aged 64 years at entry.

In the upper part of the graph in Figure 2, a hypothetical number of screen-detected cases at the first and two subsequent screenings has been shown, reflecting the experience of women starting at 64 years and repeating the test at 66 and 68 years, and thereafter leaving the programme. The excess of incidence resulting from the earlier detection will continue until the women stop having a mammogram. The decremental cases expected each year were estimated (under the assumptions of the duration and distribution of the MST) and are shown below the time axis. On this basis, it is evident that 90% of incremental cases are expected to be decremental in at least 11 years starting from the year of incidence.

It should also be noted that, contrary to what is intuitively expected, a large part of the decremental cases will have been diagnosed clinically until screening was ongoing; in our example, 65% of the incremental cases at the three screening rounds will have been decremental over the time for which the woman was continuing her screening regimen. Decremental cases that would determine the incidence rate decrease after the cessation of screening at 70 years of age are only a small proportion of the total incremental cases. Furthermore, the incidence rate decrease expected after the end of screening might be less relevant than expected because women will continue to receive mammograms outside the screening programme.

## Results

In total we included 13,519 breast cancer cases diagnosed in the pre-screening years (corresponding to years from -5 to 0). In the five years of screening (years 1 to 5), 13,999 cases were included from the six cancer registries.

Pooling the pre-screening incidence data from the six areas, the annual percentage change in the pooled incidence trend was 1.2% (95% confidence interval (CI) 0.8 to 1.6) for all breast cancers and 0.9% (95% CI 0.5 to 1.3) for invasive breast cancers only.

**Figure 3:**
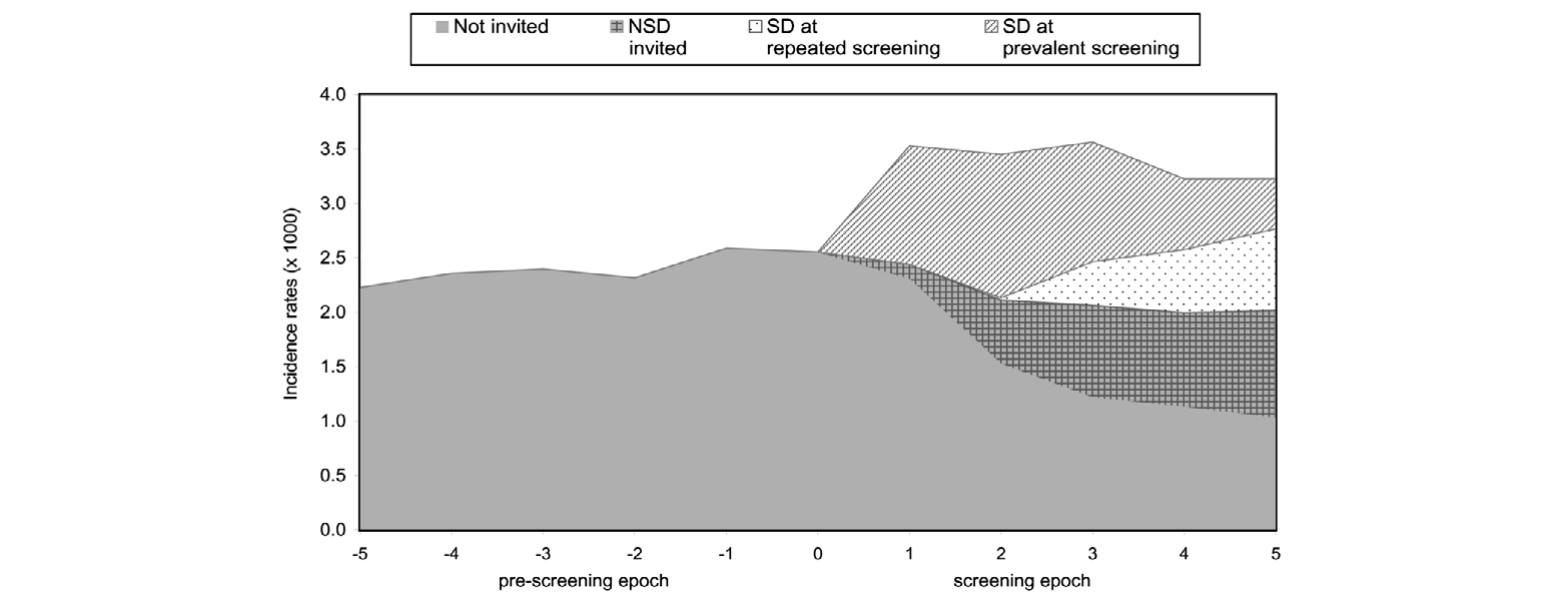
Breast cancer incidence rates by method of detection, on the screening time scale. The participants were aged 50 to 74 years. SD, screen detected; NSD, not screen detected.

Figure 3 shows the excess of incidence observed in the study screening programmes for women aged 50 to 74 at diagnosis, over the screening time scale. Population-based incidence rates are presented by method of detection (not invited and invited, divided into screen detected and not screen detected). At the end of five years from time 0, 68.2% of the 50 to 74-year-old cases (77.7% of the cases aged 50 to 69 years) had received an invitation to be screened, and 37.5% (42.8% of the cases aged 50 to 69 years) were detected at screening.

**Figure 4:**
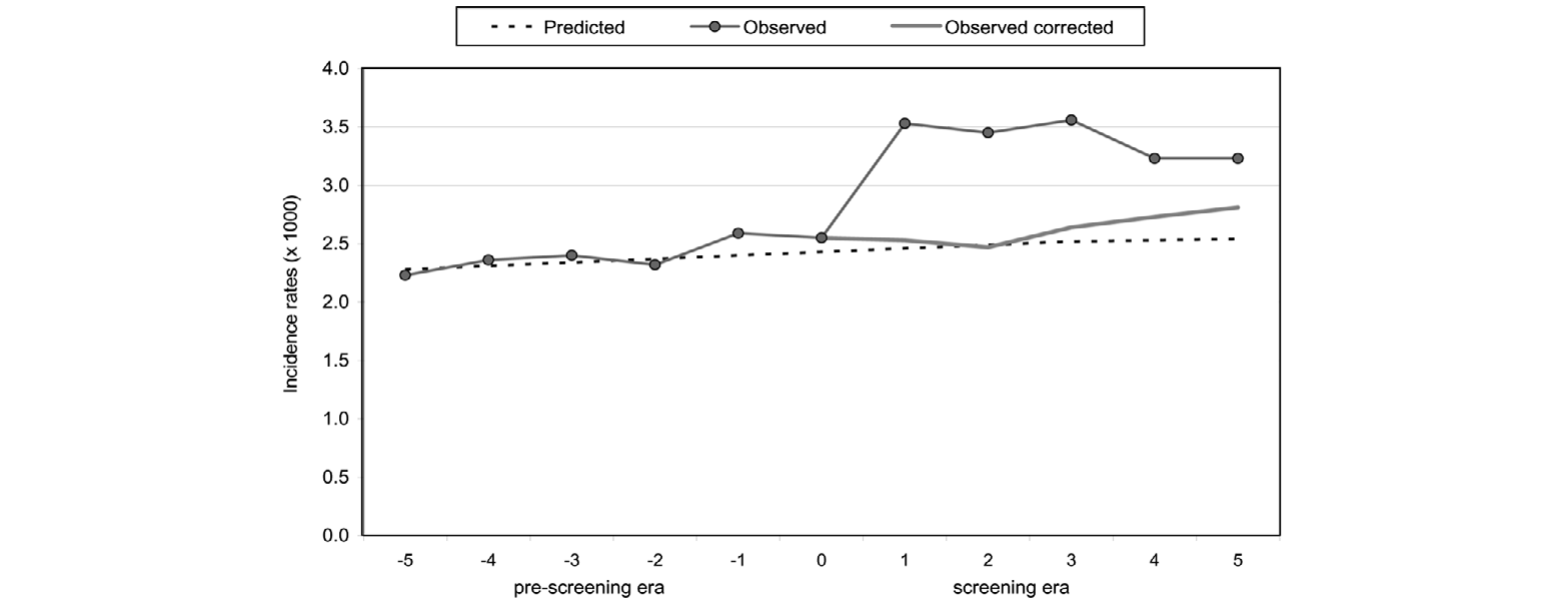
Breast cancer incidence rates predicted, observed, and observed corrected for lead time. The participants were aged 50 to 74 years.

In Figure 4 the incidence rates are presented by year relative to the start of screening and compared with the predicted rates in the absence of screening and with the data corrected for lead time. Results excluding *in situ *carcinomas were similar (data not shown).

In Table 2 the results are reported by 5-year age groups for women aged 50 to 74 years at diagnosis and compared with the predicted number of cases in the absence of screening. In total, in the first 5 years of screening, the ratio of observed to predicted *in situ *and invasive cases was 136.2% (95% CI 134 to 139%), a 36% excess of cases. After correction for lead time, the ratio for *in situ *and invasive cases was 104.6% (95% CI 102 to 107%), and for invasive cases only it was 103.2% (95% CI 101 to 106%).

**Figure 5:**
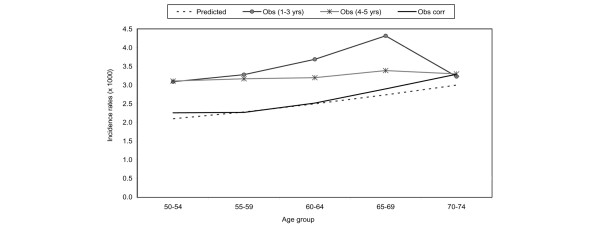
Breast cancer incidence rates by age group and years since the start of the screening.

In Figure 5 the excess of breast cancer incidence is presented by 5-year age group at diagnosis, comparing incidence rates in the period 1 to 3 years since start (this is equivalent to the period of the first round of screening) and 4 to 5 years after the start (subsequent rounds), and incidence rates after the correction for lead time. The dotted line shows the predicted incidence rates in the absence of screening. The 50 to 54-year-old women showed an excess of 7.4% in both the first and subsequent rounds. Indeed, in the period 4 to 5 years after the start, women aged 50 to 54 years were mostly new entries (prevalent screening).

**Figure 6:**
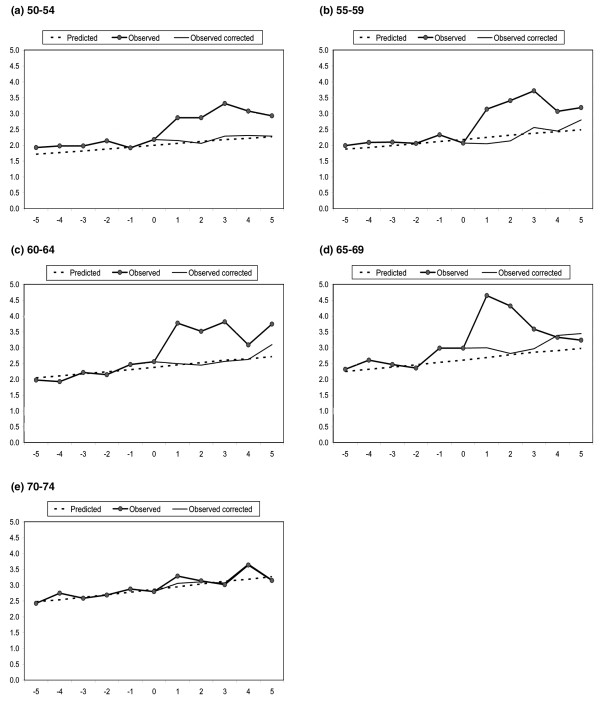
Breast cancer incidence rates predicted, observed, and observed corrected for lead time. The age groups at first invitation were as follows: **(a) **50 to 54 years; **(b) **55 to 59 years; **(c) **60 to 64 years; **(d) **65 to 69 years; **(e) **70 to 74 years.

In the following analysis, breast cancer incidence rates were estimated with a cohort approach grouped by age at the beginning of the screening programme. Women are aging over the screening time scale, so the predicted number of cases is expected to increase over time. In Figure 6 results are presented by five year age group and compared with the predicted and the corrected-for-lead-time cases. With the cohort method, the observed rates corrected for lead time corresponded very closely to the predicted ones, particularly for the 55 to 64-year-old age group. Women aged 65 to 69 years showed an increase in incidence, reaching the highest of all groups' peaks at prevalence screening. Whereas some of them could have continued screening at older ages, we observed a sharp decrease in the incidence in women within the five year follow-up period. The incidence rate decreases close to the level of the expected incidence.

## Discussion

Breast cancer service screening in Italy started in the late 1990s, and at the moment only a few areas have more than five years of follow-up. An excess of incidence was evident immediately after the start of screening and this increase was strictly related to the incremental number of screen-detected cases. The excess of incidence was especially evident in women older than 65 years at the first screening test (prevalent). This result confirms the possibility of a risk of overdiagnosis in older age groups – a risk that is higher in correspondence to the peak of breast cancer detection at prevalence screening. Incidence rates decreased at the incident screening, but they did not return to the predicted incidence rates. For women aged 70 to 74 years at the beginning of the screening programme (a group of women not involved in the screening process in the first period of screening), there was no evidence of changes in the incidence rate when comparing observed and predicted rates. The excess of incidence due to the incremental detection, which is the intended effect of screening, should correspond to a decrement of the number of cases arising in subsequent years. However, as we have shown in the example of women aged 64 years at their first screening and continuing to receive screening until 69 years old, the decrement in the number of excess cases starts immediately and would occur until screening is ongoing. It could therefore not be seen in the observed rates.

To document the residual excess of cases (that is, the overdiagnosis due to screening) follow-up studies should extend over many years from the start of a programme. However, in the absence of a control and given the increasing uncertainty over time in the prediction of long-term incidence trends, evaluation is difficult.

MacCann and colleagues [11] have highlighted the peak of incidence observed in the 60 to 64-year-old age group at prevalent screening in the UK programme and the subsequent decline in the observed incidence about six years after the start. An approach taking into account lead time and using a model correcting observed data for lead time was used by Paci and colleagues [8] in the evaluation of the excess of incidence and overdiagnosis in the Florence City programme. In that analysis, the probability of cases being detected before or after the study period was estimated. The method of quantification used in the present paper is similar, but we have modelled the decremental number of cases over the years after the screen detection.

Randomised population-based trials have recently been reviewed by Moss [12], suggesting no overdiagnosis related to the incident screening and an excess of 10 to 15% at seven to eight years of follow-up from the start in trials with no control group screening. Data on randomised trials have been updated by the recent results published by the Malmö, Goteborg and Two County studies [13-15]. The two Swedish studies confirmed the absence of excess of incidence in trials in which the control group was screened at the end of the study period. In their analysis, taking into account the statistical adjustment for lead time, Duffy and colleagues showed a risk of overdiagnosis lower than 5%.

The Malmö trial showed, at the end of 15 years of follow-up after the study's end, a residual excess equal to 10% of cases in the screened group [13]. There were two major problems in the evaluation of the long-term residual excess of cases in the screened arm. First, because 60% of the women died in the Malmö study, there is the need to assess the impact of competing causes of death on the overdiagnosis estimate. Second, women could have mammograms within or outside the programme (including after the last age for screening and differential screening practice between groups), so it is difficult to assess the excess over such a long period.

The statistical modelling used in this paper is based on two major assumptions. The first is that the predicted trends can be estimated from pre-screening data. The second assumption concerns the estimation of sojourn time and its exponential distribution, and, thus, of the lead time. There is much evidence supporting the estimates we have adopted in this paper, and models for the assessment of breast cancer screening have been shown to be quite consistent in predicting observed results. Recent modelling of breast cancer screening with several statistical models has confirmed the consistency of our knowledge of the natural history of the disease [14].

However, estimates are always subject to criticism. For these reasons we performed a sensitivity estimate. With the upper limit (4.8 years) of 95% CI of the lead time estimate for 50 to 74-year-old women, the estimate of overdiagnosis was 102.8, including the *in situ *carcinomas; that is, an excess of 3%. To quantify further the residual excess of incidence, namely the exclusion of overdiagnosis, the lead time needed to equalise the predicted and corrected-for-lead-time cases (an observed:expected ratio of 1) was calculated as 6.0 years. This value is too long for the current estimates, although a similar value was recently estimated in the Norway screening programme [15].

Birth cohort approach has been shown by Moller and colleagues [16] as the more informative approach to demonstrate the decrease in the incidence rate after the end of the screening regimen. In the large programmes of North Europe, as in our study, a smaller number of women continue with screening when they are over the age of 70 years.

The cohort analysis comparing the predicted with the corrected-for-lead-time cases confirmed the possible risk of overdiagnosis for women having a mammogram aged 65 to 69 years at entry. This age group showed the highest peak of incidence at prevalent screening.

The strength of this evaluation of the impact of breast cancer screening lies in its study of population-based characteristics and from the collection of individual screening histories. In most studies published so far, results have been estimated from aggregated data and without the possibility of a subject-specific attribution of the individual lead time. This, along with the methodological difference related to the probabilistic modelling in our paper of the postponement of cases, might account for the difference between our findings and those of Jonsson and colleagues [17], who showed a minimal change in the excess of incidence after correction for lead time. There is also the possibility of confounding with other effects on incidence in the paper by Jonsson and colleagues, as acknowledged in their discussion.

## Conclusion

In the screening age groups (ages 50 to 74 years) the excess of incidence was 36.2% and after correction for lead time the remaining excess of *in situ *and invasive carcinomas was 4.6% (95% CI 2 to 7%), less than 5%. Excluding *in situ *carcinomas the excess was 3.2% (95% CI 1 to 6%).

Longer follow-up is needed to confirm this estimate, but at five years since the programme started, the risk of overdiagnosis is modest, considering that it is not possible at the moment to distinguish on an individual basis which cancer will progress and which will not. Further research is needed to improve our understanding of the markers of tumour progression and so enhance our ability to avoid over-treatment of screen-detected cases.

## Abbreviations

CI = confidence interval; MST = mean sojourn time.

## Competing interests

The authors declare that they have no competing interests.

## Authors' contributions

EP was the project leader and with GM designed the study and with DP made the analysis and drafted the manuscript. All authors were responsible for data analysis. PB, VDL, FF, CC, SF, LM, ACF, SR, NS, FS, AT, RT and MZ organised the local fieldwork and data collection in the IMPACT screening centres and cancer registries. All authors participated in the planning of the study, in the intermediate evaluation and in the final revision of the manuscript.

## Figures and Tables

**Table 1:** Pre-screening and screening time periods of the study, and number of breast cancer cases

Register	Study period	Start year of screening	Pre-screening cases	Screening cases	Total cases
Torino	1986–1996	1992	3,017	3,185	6,202
Parma	1991–2001	1997	1,567	1,714	3,281
Ferrara	1991–2001	1997	1,539	1,400	2,939
Modena	1989–1999	1995	2,283	2,056	4,339
Romagna	1990–2000	1996	3,029	3,405	6,434
Firenze	1986–1995	1991	2,084	2,239	4,323
Totals			13,519	13,999	27,518

**Table 2:** Observed, predicted, screen-detected breast cancer cases and observed corrected-for-lead-time cases, by age group

Age group (years)	O	P	O/P	Screen-detected	O_c_	O_c_/P	95% CI, Oc/P
50–54	1,957	1,328.1	147.4	749	1,426	107.4	102–113
55–59	2,017	1,418.8	142.2	950	1,410	99.4	94–105
60–64	2,192	1,555.8	140.9	995	1,566	100.7	96–106
65–69	2,340	1,605.8	145.7	1,031	1,697	105.7	101–111
70–74	1,788	1,646.9	108.6	127	1,807	109.7	105–115
50–74	10,294	7,555.3	136.2	3,852	7,906	104.6	102–107

O, observed; P, predicted; O_c_, observed corrected for lead time; CI, confidence interval.
